# Using cortical organoids to understand the pathogenesis of malformations of cortical development

**DOI:** 10.3389/fnins.2024.1522652

**Published:** 2025-01-15

**Authors:** Kellen D. Winden, Isabel Gisser, Mustafa Sahin

**Affiliations:** Department of Neurology, Rosamund Stone Zander Translational Neuroscience Center, Boston Children’s Hospital, Harvard Medical School, Boston, MA, United States

**Keywords:** ASD, tuberous sclerosis, PTEN hamartoma tumor syndrome, iPSCs, cortical organoids, mTOR

## Abstract

Malformations of cortical development encompass a broad range of disorders associated with abnormalities in corticogenesis. Widespread abnormalities in neuronal formation or migration can lead to small head size or microcephaly with disorganized placement of cell types. Specific, localized malformations are termed focal cortical dysplasias (FCD). Neurodevelopmental disorders are common in all types of malformations of cortical development with the most prominent being refractory epilepsy, behavioral disorders such as autism spectrum disorder (ASD), and learning disorders. Several genetic pathways have been associated with these disorders from control of cell cycle and cytoskeletal dynamics in global malformations to variants in growth factor signaling pathways, especially those interacting with the mechanistic target of rapamycin (mTOR), in FCDs. Despite advances in understanding these disorders, the underlying developmental pathways that lead to lesion formation and mechanisms through which defects in cortical development cause specific neurological symptoms often remains unclear. One limitation is the difficulty in modeling these disorders, as animal models frequently do not faithfully mirror the human phenotype. To circumvent this obstacle, many investigators have turned to three-dimensional human stem cell models of the brain, known as organoids, because they recapitulate early neurodevelopmental processes. High throughput analysis of these organoids presents a promising opportunity to model pathophysiological processes across the breadth of malformations of cortical development. In this review, we highlight advances in understanding the pathophysiology of brain malformations using organoid models.

## Introduction

Malformations of cortical development include a variety of abnormalities from widespread migration defects to highly restricted areas of cerebral dysgenesis (Barkovich et al., 2012; Desikan and Barkovich, 2016). Early head growth is dependent on the expansion of the brain, and therefore, global defects in the coordination of neural development cause reduced head size or microcephaly. There has been substantial progress in understanding the genetic underpinnings of these disorders, which has implicated several molecular processes involved in specific patterns of abnormal cortical development. For example, several genes associated with mitotic spindle formation and cell division—such as *ASPM* and *CENPJ*—have been linked to microcephaly, which is thought to be due to defects in neurogenesis (Thornton and Woods, 2009; Degrassi et al., 2019). The microtubule network is critically involved in regulating cellular morphology, and impaired migration of neural progenitors or immature neurons leads to disorganized cortical layering (Kato and Dobyns, 2003; Moon and Wynshaw-Boris, 2013). These disorders can also display various patterns of abnormalities in gyration and cortical thickness on brain imaging. Some migration disorders such as periventricular nodular heterotopia caused by variants in *FLNA* are associated with the presence of unmigrated cortical tissue in the ventricular zone and multiple neurological symptoms (Loft Nagel et al., 2022). In addition, there is growing appreciation of other cellular processes such as the role of the endoplasmic reticulum in neuronal migration, highlighted by the identification of genes such as *LNPK*. The encoded protein, lunapark, stabilizes ER junctions, and *LNPK* mutations are linked to a variety of neurological conditions with symptoms including hypoplasia of the corpus callosum and epilepsy (Accogli et al., 2023). While these disorders typically lead to broad involvement of the cortex, several other genetic variants cause developmental abnormalities only in specific areas.

This subset of cortical malformations, known as focal cortical dysplasias (FCDs), often only involves localized areas of the cortex. Clinically, FCDs have been classified into three broad categories: FCD type I and III, which are often associated with secondary disorders such as injury and tumors, and FCD type II, which has been linked to several genetic disorders (Najm et al., 2022). More specifically, FCD type II is associated with abnormalities in regulation of the mechanistic target of rapamycin (mTOR), a central kinase involved in cell growth and proliferation (Ljungberg et al., 2006). One disorder associated with both FCD and mTOR signaling is Tuberous Sclerosis Complex (TSC), an autosomal dominant genetic disorder caused by variants in the *TSC1* or *TSC2* genes (Salussolia et al., 2019). These genes encode the proteins hamartin and tuberin, which form a complex to negatively regulate mTOR (Switon et al., 2017). In addition to its symptomatology, TSC exemplifies a common feature of FCD-associated disorders – the difficulty of modeling them faithfully in animals. Patients with TSC carry heterozygous variants in *TSC1 or TSC2*, yet heterozygous animal models display subtle or no phenotypes (Uhlmann et al., 2002; Kirschstein, 2012; Yuan et al., 2012). Among TSC animal models that do develop FCD-like brain lesions, most do not exhibit a seizure phenotype or distinct behavioral symptoms, which mitigates their translational utility (Way et al., 2009; Feliciano et al., 2012). Unfortunately, these results are relatively common among several malformations of cortical development, which implies that their pathogenesis arises from dysfunction of human-predominant processes in brain development.

Thus far, the study of human brain development has been limited by the lack of access to fetal tissue. However, with recent improvements in stem cell technologies, human pluripotent stem cells can now be used to study early developmental processes (Saha and Jaenisch, 2009). Significant progress made in reprogramming and gene editing techniques that allow for generation of induced pluripotent stem cells (iPSCs) from patients carrying specific genetic variants (Soldner and Jaenisch, 2018). These iPSCs, in turn, can be differentiated into neurons in culture. This technique enables researchers to study the effects of highly specific genetic alterations on neuronal differentiation and function *in vitro*. Currently, investigators use either 2-dimensional or 3-dimensional cell culture systems for their studies (Engle et al., 2018). 2-dimensional systems are typically more accessible for certain experimental questions, but they lack the complexity, organization, and cell–cell interactions seen in the living brain. Therefore, in recent years, researchers have prioritized developing 3-dimensional cell culture systems, commonly known as organoids, to recapitulate early human brain development (Lancaster et al., 2013; Pasca et al., 2015; Qian et al., 2016; Quadrato et al., 2017; Pasca, 2018; Velasco et al., 2019). Well-established protocols allow researchers to generate region-specific organoids, which mirror developmental patterning seen in the developing brain (Pasca et al., 2015; Gordon et al., 2021). Furthermore, fusing differently patterned organoids into “assembloids” may elucidate complex interactions between disparate brain regions (Pasca, 2019). These emerging technologies present a promising opportunity to understand mechanisms of early brain development (Di Lullo and Kriegstein, 2017; Qian et al., 2019). This review will explore recent advancements in organoid technology and the utility of 3-dimensional systems for exploring the mechanisms of malformations of cortical development.

## Organoid models and brain development

Self-organizing three-dimensional aggregates of pluripotent stem cells have become to be known as organoids (Pasca et al., 2022). Early organoid technologies allowed stem cells to aggregate and differentiate spontaneously with minimal direction (Kadoshima et al., 2013; Lancaster et al., 2013; Quadrato et al., 2017). These organoids developed a myriad of cell fates, including neurons and glia that showed variable levels of maturity, as well as non-neural tissue. Remarkably, neurons within these organoids were spontaneously active, demonstrated functional connections, and were responsive to physiological stimuli (Lancaster et al., 2013; Quadrato et al., 2017). Following these initial protocols, numerous techniques have since been published which make use of patterning factors to drive specific, regionalized cell fates (Muguruma et al., 2015; Pasca et al., 2015; Sakaguchi et al., 2015; Qian et al., 2016; Velasco et al., 2019). This strategy reduces variability between organoids, making them a more reproducible model system (Velasco et al., 2019). Emerging technologies using microfluidics to mimic growth factor gradients may be able to further refine organoid models by inducing patterning along rostral-caudal or dorsal-ventral axes (Rifes et al., 2020; Pallavicini et al., 2024; Xue et al., 2024). Furthermore, organoids have been shown to display signatures at the DNA, RNA, and protein levels that correlate with human cortical development. Transition mapping of RNA sequencing data from cortical organoids and human cortex samples has shown that gene expression changes seen *in vitro* align with both prenatal and postnatal stages of human cortical development (Gordon et al., 2021). Additionally, single-cell RNA sequencing has revealed transcriptomic similarities between fetal cortical tissue and cortical organoids, specifically, significant overlap in transcripts related to extracellular matrix gene expression, transcription regulation, glial delamination, and neurite outgrowth (Camp et al., 2015). Organoids also bear epigenetic similarities to human tissue (Zenk et al., 2024). Cortical organoids and fetal brain tissue showed overlapping methylation signatures that corresponded to super-enhancers (Luo et al., 2016). However, organoids and brain samples demonstrated distinct clusters of methylation, suggesting persistent differences between *in vitro* and *in vivo* tissue (Luo et al., 2016). Despite these methylome differences, cortical organoids bear similarity to the fetal brain in regard to the activity of histone modifiers, which suggests these model systems may yet be used to understand epigenetic control of development (Gordon et al., 2021). Finally, mass spectrometry has revealed a 40% overlap in proteomic identity between 45-day-old cortical organoids and fetal brain tissue ranging from gestational weeks 16–20 (Nascimento et al., 2019). Enriched pathways included metabolic processes, cell–cell adhesion, cortex development, cytoskeleton, axonal transport and outgrowth, and neuron projection development. Improvements to existing protocols will ideally increase the overlap between the genomic and proteomic profiles of organoids and fetal tissue. However, even with their present validity, organoid methods present a promising opportunity to address questions related to fetal brain development.

## Organoid models of microcephaly and associated cortical defects

Many genes have been associated with microcephaly, and microcephalic patients may or may not have other abnormalities in cortical development. Primary microcephaly is typically observed at birth, and genetics studies have demonstrated that a majority of the genes associated with this disorder are associated with centriole biogenesis (Jayaraman et al., 2018). The centriole is a barrel-shaped protein complex that is necessary for mitotic spindle formation during replication and localizes to the primary cilium during quiescence (Bornens, 2012). Studies in animal models have demonstrated that several genes associated with primary microcephaly cause instability or reduced number of centrosomes, which leads to impairments in neurogenesis (Barrera et al., 2010; Jayaraman et al., 2016). However, the degree of microcephaly and the cognitive phenotypes associated with disruption of these genes are far less than what is observed in human patients (Pulvers et al., 2010; Fujimori et al., 2014). These data suggest that while primary microcephaly genes participate in similar cellular pathways in animal models, human brain development utilizes these processes differently from rodent brain development and is substantially more susceptible to their disruption. Therefore, investigators have begun using organoid models to understand the interactions between centriole biology and the pathogenic mechanisms in abnormal brain development.

Organoids generated from stem cells carrying pathogenic variants in several primary microcephaly genes, including *ASPM*, *CDK5RAP2*, *CENPJ*, *CIT*, *KATNB1*, and *WDR62*, have been shown to be smaller, consistent with the human phenotype (Lancaster et al., 2013; Jin et al., 2017; Li et al., 2017; An et al., 2022; Dell'Amico et al., 2023; Pallavicini et al., 2024). As in studies of other cell types, the affected proteins were typically localized to the centrosome within organoids, and CENPJ variants were found to cause reduced distance between centrioles (An et al., 2022). The decreased size of these organoids has been attributed to both reduced cell proliferation and increased cell death, and increased dsDNA breaks and P53 activation have been reported, suggesting potential disease mechanisms (An et al., 2022; Pallavicini et al., 2024). There are typically decreased numbers of neuroprogenitors and immature neurons (Lancaster et al., 2013; Jin et al., 2017; Li et al., 2017; An et al., 2022). Finally, neurons from these organoids have been shown to have decreased spontaneous activity (Li et al., 2017). These data demonstrate impairments in the molecular coordination of cell division lead to global defects in the formation of the cerebral cortex. However, further studies are necessary to explain why human brain development is more sensitive to centrosomal abnormalities than many model organisms.

Neuronal migration defects have often been implicated in microcephaly, as well as other disorders such as lissencephaly, polymicrogyria, and periventricular nodular heterotopia. Studies of genes associated with lissencephalies have identified several potential mechanisms (Romero et al., 2018). The best studied gene, *Pafah1b1*, has been shown to play a critical role in nuclear and centrosomal movement through its interaction with dynein during cellular migration (Tsai et al., 2007). Humans with heterozygous loss of function variants in *PAFAH1B1* display an abnormal four layered cortex with the presence of under-migrated neurons (Friocourt et al., 2011). However, most *Pafah1b* heterozygous animals do not show any cortical abnormalities, although disruption of the second allele of *Pafah1b1* does interfere with neuronal migration (Hirotsune et al., 1998; Cahana et al., 2001). In contrast, there are examples such as the homeobox transcription factor, *ARX*, where loss in both humans and mice leads to alterations in cortical development and epilepsy (Kato et al., 2004; Colasante et al., 2015). These data suggest that human brain development is more sensitive to haploinsufficiency in genes involved in neuronal migration than mouse brain development, although some genetic defects result in similar phenotypes across species.

In contrast to the centrosome-associated disorders above, deficits in radial glia positioning and orientation are frequently observed due to disruption of genes associated with lissencephaly, suggesting that this population of cells is particularly affected (Bershteyn et al., 2017; Iefremova et al., 2017; Klaus et al., 2019; Fair et al., 2023; Wang et al., 2023; Geng et al., 2024; Werren et al., 2024). One characteristic feature of cortical organoids is the formation of rosettes with a layer of neuroprogenitors, similar to the ventricular zone in the developing brain. Radial glia typically extend fibers perpendicular to this structure that serve as scaffolds for migration, and these projections have been shown to be disorganized or absent due to several genetic variants (Bershteyn et al., 2017; Iefremova et al., 2017; Klaus et al., 2019; Wang et al., 2023). In addition, studies have demonstrated abnormalities in cell division of neuroprogenitors (Iefremova et al., 2017; Werren et al., 2024), as well as impaired migration of immature neurons (Bershteyn et al., 2017; Klaus et al., 2019). Outer radial glia (oRG) are a population of progenitors that are dramatically expanded in human brain development, and studies have developed methods to reliably study this cell type in organoids (Watanabe et al., 2017; Andrews et al., 2023; Walsh et al., 2024). Remarkably, one study demonstrated that oRG are particularly affected in organoids with deletion of *PAFAH1B1*, suggesting an explanation why animal models may not recapitulate phenotypes observed in patients (Bershteyn et al., 2017). Interestingly, abnormalities in Wnt/B-catenin signaling have been found to be associated with multiple genetic variants, although there have been some conflicting observations regarding directionality of the changes (Iefremova et al., 2017; Fair et al., 2023; Geng et al., 2024). Taken together, these studies suggest primary involvement of radial glia, with potential specificity for the human-enriched oRG, in malformation disorders that are caused by abnormalities in neuronal migration. Further studies are needed to understand whether the sensitivity of human cells to haploinsufficiency of these genes is due to role of these proteins in specific cell types such as oRG or complex regulatory mechanisms that require the presence of both alleles for full expression.

Organoid studies have also provided some new perspectives on mechanisms involved in brain malformations. For example, a screen for genes involved in brain organoid growth identified *IER3IP1*, which is predicted to be localized to the endoplasmic reticulum. In organoids with *IER3IP1* deletion, there was increased ER stress and abnormal ER morphology (Esk et al., 2020). A second study was searching for genes involved in interneuron migration using assembloids, and the investigators identified *LNPK* as a key mediator of this process. Further studies demonstrated that this protein was critically involved in ER movement that precedes saltatory migration of immature inhibitory neurons (Meng et al., 2023). In addition, metabolic processes have also been implicated in cortical development. Organoids with variants in *PNPLA8*, which encodes a lipid phosphatase, also showed impairments in oRG. In addition, these organoids demonstrated alterations in lipid composition, and supplementation with one of these components could partially rescue some of the cellular phenotypes (Nakamura et al., 2024). Another gene involved in lipid metabolism, *FASN*, also showed radial glia abnormalities (Gonzalez-Bohorquez et al., 2022). These data highlight the possibility for identifying novel mechanisms of malformations of cortical development primarily using human models.

## Organoid models of focal cortical dysplasias

mTOR is a central kinase that is involved in regulation of cellular metabolism and growth in response to multiple metabolites, growth factors, and other inputs. Multiple upstream proteins have been shown to regulate mTOR, and dysfunction of several of these factors has been shown to play a role in the formation of FCD type II (Winden et al., 2015). TSC is prototypical among these disorders and leads to the formation of cortical tubers in most patients. Pathologically, cortical tubers are identical to focal cortical dysplasia type IIb, which are characterized by dyslamination, presence of abnormal cell types, and astrogliosis (Boer et al., 2008; Muhlebner et al., 2016). These lesions can be visualized on prenatal MRI demonstrating that they are inextricably linked to the development of the brain. In addition, while their appearance on imaging can change throughout the lifetime, these lesions do not demonstrate appreciable growth, distinguishing them from other tumors that occur in TSC (Peters et al., 2015). Heterozygous animal models of TSC typically display subtle or no neurological phenotypes, contrasting with the human disorder (Uhlmann et al., 2002; Kirschstein, 2012; Yuan et al., 2012). Despite this, molecular and cellular phenotypes such as mTOR hyperactivation, cytomegaly, and increased neurite branching are typically observed with deletion of the second allele of either *Tsc1* or *Tsc2* (Meikle et al., 2007; Way et al., 2009; Feliciano et al., 2012; Yuan et al., 2012). While lesions seen in these rodent models bear similarity to human cortical dysplasias, none have all of the features that define cortical tubers, suggesting the presence of other pathological mechanisms. These data are consistent with other brain malformation disorders discussed above where heterozygous humans are more susceptible to neural dysfunction compared to heterozygous rodents. However, the specific mechanism may not only involve sensitivity to haploinsufficiency but also somatic variants leading to loss of heterozygosity (Crino et al., 2010). Given the dramatically expanded size of the human cortex, it is possible that somatic variants may play a greater role in human disease compared to model organisms. These data have led investigators to examine both the heterozygous and homozygous loss of the *TSC1* and *TSC2* genes in human cells.

The first study of cortical organoids in TSC demonstrated that second hit mutations in either *TSC1* or *TSC2* were necessary for hyperactivation of mTOR signaling and led to impaired neurogenesis and increased gliogenesis (Blair et al., 2018). In addition, they demonstrated that induced second hit mutations caused formation of cells that were similar to abnormal cells that are present in cortical tubers and FCD type IIb (Blair et al., 2018). A different study of TSC organoids found that heterozygous iPSCs formed organoids with increased prevalence of interneuron progenitors typically derived from the caudal ganglionic eminence, which are associated with increased EGFR signaling (Eichmuller et al., 2022). In addition, they proposed that these cells had similarities to both cortical tubers and subependymal nodules, which are precursors to astrocytomas, and they concluded that second hit mutations are not necessary for abnormalities in TSC (Eichmuller et al., 2022). It is unclear why these two studies arrived at directly contradictory interpretations of their data, but one important difference between these studies is that they used different patterning protocols, which may account for some of the discrepancies if they were evaluating different regions within the neuraxis.

*PTEN* is very similar to *TSC1/2* in that it is also an upstream negative regulator of mTOR, and heterozygous variants lead to *PTEN* Hamartoma Tumor Syndrome (PHTS). PHTS encompasses a group of rare syndromes with similar clinical features, including Cowden’s syndrome, Bannayan-Riley-Ruvalcaba syndrome, Lhermitte-Duclos disease, and Proteus-like syndrome (Pilarski et al., 2013). As seen with *TSC1/2* mutations, mutation of *PTEN* results in increased mTORC1 activity and increases risk for certain cancers and hamartomas. Patients also often present with ASD and developmental delay (Winden et al., 2018). However, PHTS pathology diverges from TSC in regard to cortical malformations. PHTS is often associated with white matter abnormalities and general disorganized cortical development (i.e., heterotopia, polymicrogyria) rather than distinct tubers as seen in TSC (Shiohama et al., 2020; Shelkowitz et al., 2023). As seen in TSC, *PTEN*+/− animals demonstrate subtle changes (Page et al., 2009; Smith et al., 2016), while loss of the second allele leads to neuronal abnormalities and abnormal behavior (Kwon et al., 2006). Organoids with heterozygous *PTEN* variants have been shown to have deficits in generating mature cortical neurons, which is associated with increased numbers of outer radial glia (Pigoni et al., 2023; Kang et al., 2024). Radial glia were also abnormally oriented surrounding ventricular zone-like structures. Interestingly, deletion of the second allele of *PTEN* did not have a strong effect on differentiation. *PTEN*+/− organoids demonstrated spontaneous hyperactivity, consistent with its association with epilepsy and ASD (Dhaliwal et al., 2024). In addition, *PTEN+/−* immature neurons displayed decreased sodium current and Nav1.1 expression, suggesting a potential mechanism for the abnormalities in activity and providing a potential link between abnormal cellular development and seizure activity in PTHS (Kang et al., 2024).

Another example of FCD related to mTOR disinhibition is polyhydramnios, megalencephaly, symptomatic epilepsy (PMSE) syndrome secondary to biallelic loss of function of *STRADA*. *STRADA* forms a complex upstream of the TSC1/2 complex and loss of this pathway leads to unregulated mTOR activity. Interestingly, organoids with biallelic *STRADA* variants showed reduced cortical neurogenesis and increased out radial glia (Dang et al., 2021), suggestive of the phenotypes observed in TSC and PHTS described above. It is interesting to note that these disorders define a spectrum from highly localized pathology in TSC to broad involvement in PMSE with similar abnormalities in molecular and cellular pathogenic mechanisms. Together, these data show that the specific developmental window and fraction of cells affected by mTOR disinhibition is critical for understanding the consequences for brain development.

## Future directions

These studies provide an exciting foundation for understanding the pathogenesis of malformations of cortical development using human 3D models. Unsurprisingly, many pathogenic mechanisms converge onto processes that occur early in development with neuroprogenitors and radial glial being the most commonly affected cell types. Given the differences in brain development between humans and other model organisms, there are several pathways that are functionally different in humans (Lui et al., 2014; Andrews et al., 2020). With advances in 3D human models, these mechanisms are becoming tractable experimentally. However, one difficulty with these models has been variability, which has contributions from several areas, but studies have shown that the initial state of the stem cell lines from which they are generated is critically important (Watanabe et al., 2022; Glass et al., 2024; Sandoval et al., 2024). As strategies for mitigating these sources of variability are identified, they will enable more detailed study of pathogenic changes in molecular and developmental pathways. For example, understanding the mechanisms that lead to formation of the diversity in radial glia will undoubtedly lead to new insights into these malformation disorders. Another limitation is that the lack of a vasculature system leads to poor nutrient distribution within the organoid, which can result in metabolic stress and impair cell type specification (Bhaduri et al., 2020; Uzquiano et al., 2022). Finally, the pace and level of maturity of these 3D models limits study of mature neuronal networks. Most studies examine organoids corresponding to mid-fetal brain development, but most neurological disorders do not present prenatally. Even in the malformation syndromes discussed above that cause early defects in brain development, many symptoms that contribute to morbidity in these disorders do not present until later. Therefore, studying more mature neuronal networks will be imperative to furthering insights into these disorders. iPSC-derived neuron transplantation into rodent models has been demonstrated to substantially facilitate neuronal maturation, and therefore, this technique could be invaluable to understanding these processes that occur later in development (Linaro et al., 2019; Revah et al., 2022). In addition, it has been shown that epigenetic maturation is slower in human neurons compared to other species and that strategies to increase the rate of epigenetic change facilitate neuronal maturation (Ciceri et al., 2024; Hergenreder et al., 2024). Metabolic maturation is also slower in human neurons, and facilitating this process led to increased dendritic complexity and enhanced activity dependent responses, characteristic of increased neuronal maturity (Iwata et al., 2023). These and other techniques will aid understanding pathogenic mechanisms in brain development, which will provide platforms to identify novel therapeutic strategies for these disorders.
